# A Screen for Key Genes and Pathways Involved in High-Quality Brush Hair in the Yangtze River Delta White Goat

**DOI:** 10.1371/journal.pone.0169820

**Published:** 2017-01-26

**Authors:** Haiyan Guo, Guohu Cheng, Yongjun Li, Hao Zhang, Kangle Qin

**Affiliations:** Key Laboratory for Animal Genetics & Molecular Breeding of Jiangsu Province, College of Animal Science and Technology, Yangzhou University, Yangzhou, China; China Agricultural University, CHINA

## Abstract

The Yangtze River Delta White Goat is the only goat breed that produces high-quality brush hair, or type III hair, which is specialized for use in top-grade writing brushes. There has been little research, especially molecular research, on the traits that result in high-quality brush hair in the Yangtze River Delta White Goat. To explore the molecular mechanisms of the formation of high-quality brush hair, High-throughput RNA-Seq technology was used to compare skin samples from Yangtze River Delta White Goats that produce high-quality hair and non high-quality hair for identification of the important genes and related pathways that might influence the hair quality traits. The results showed that 295 genes were expressed differentially between the goats with higher and lower hair quality, respectively. Of those genes, 132 were up-regulated, 62 were down-regulated, and 101 were expressed exclusively in the goats with high-quality brush hair. Gene Ontology and Metabolic Pathway Significant Enrichment analyses of the differentially expressed genes indicated that the *MAP3K1*, *DUSP1*, *DUSP6* and the MAPK signaling pathway might play important roles in the traits important for high-quality brush hair.

## Introduction

The Yangtze River Delta White Goat is the unique goat species that can produce high-quality meat, skin, and Type III hair. Brush hair is usually divided into three grades, Type I low-quality hair, Type II mid-quality hair, and Type III high-quality hair[1], which is specialized for making top-grade writing brushes and has many characteristics such as white color, straight peak, fine luster, and rich elasticity. The Yangtze River Delta White Goat, also called the Haimen goat, is found mainly in the Yangtze River delta plain, the central area of which is located in Haimen County, Nantong, Jiangsu Province[2]. It also has a laudatory name, brush hair goat, for its special production of hair. With a price up to 2,500~3,000 US dollar per kg, Type III hair is popular in Japan, Korea, Singapore, and other Southeast Asian countries influenced by Chinese culture and usually a traditional Chinese export in tight supply[3].

Type I hair produces coarse hair, its surface scales resemble fish scales, and its hair-shaft center contains a large number of medulla. Type II hair also produces coarse wool, its medullary cavity is small, with relatively few medulla. Type III hair is made up of cuticula and cortex. The hair-shaft center contains a small amount of medulla. Type III hair has the characteristics of fine wool, heterotypical hair, and coarse hair. The cuticula consist of circular scales, hybrid scales, and fish-scale scales. The upper part of the medullary cavity consists of transparent or spotty marrow. The middle of the medullary cavity is fine and contains fewer medulla. The under part of the medullary cavity has a large number of medulla[4]. Type III hair grows only on the back and neck ridge of the animal, whereas Type I and Type II hair are widely distributed[4]^.^ Non high-quality brush hair refers to Type I and Type II hair. High-quality brush hair refers particularly to Type III hair, which is a unique product of the Yangtze River Delta White Goat[5].

The hair root from outside to inside is made up of the dermal sheath, the outer root sheath, and the inner root sheath (IRS). The IRS determines the character of the hair shaft. The IRS proliferates upward inside of the outer root sheath to support the growth of the fiber. In the sebaceous-glands layer, the IRS cells degenerate into pieces and finally disappear, releasing the hair fiber[6]. The production of brush hair is seasonal, which might be related to the growth cycle of the hair follicles[7]. The hair follicle growth cycle includes anagen, catagen, and telogen[8]. Cytokines play an important role in the regulation of the hair follicle growth cycle. Hair follicle stem cells have a certain life cycle, and the hair follicle subsequently enters catagen, the split termination[9]. The hair follicle growth and development process involves many genes and signaling molecules. Most of those signaling molecules belong to the Wingless-related (Wnt) pathway, Sonic Hedgehog (Shh) pathway, MAPK pathway, and so on[10–11]. Previous studies have indicated that the MAPK pathway plays an important role in the growth and development of hair follicles[12–14]. The MAPK pathway can regulate cell growth, differentiation, and apoptosis and can participate in the regulation of hair-follicle growth and development[15–16]. In this study we used RNA-Seq technology to explore the important genes and pathways related to the formation of type III hair. The annotation of Unigenes from both the high hair quality and the non high hair quality phenotypes was obtained, and a gene pool containing all of the differentially expressed genes of the metabolic pathways was established to provide supporting evidence for the further study of the molecular mechanisms underlying the Type III hair traits in the Yangtze River Delta White Goat.

## Materials and Methods

### Ethics statement

The animal experiments were carried under the regulation of Yangzhou University Ethics Committee, and we have attached the Certificate on Animal experiments in this study (the certificate in the form of Supporting Information attached at the end of article), which is approved by Yangzhou University Ethics Committee. I confirm that the field studies did not involve endangered or protected species. This study location in the Haimen State Haimen Goat Farm and key laboratory for animal genetics and molecular breeding of Jiangsu Province, college of animal science and technology, Yangzhou University, and we have got their permission. I confirm that I received permission in advance of the study being conducted from your local ethics committee, and this permission for this specific study. The animal care and use were fully compliant with national standards of China. We followed the protocols and no animals were sacrificed for the purposes of this study. We followed the management and regulations for the goats. Rams were fed alone, and ewes fed together, and each goat occupied the living space of more than 1 square meters. The goat shed have the good condition of ventilation and penetrating light. We fed it once a day with free drinking water. We cleared the feces and dirt every day and disinfected the shed once a week. After operations, the skin cuts of the experimental animals were disinfected and cared well to restore.

### Preparation of test samples

The experiments of goats came from the Haimen State Haimen Goat Farm. Six half-sib rams of 6~8 months were selected, including three high-quality hair and three non high-quality hair. From each goat, about 2 cm^2^ of skin along the cervical spine was sampled and cryopreserved. The goats were given local anesthesia when the samples were taken. The six samples were divided into two groups: group A, comprising the samples from the three high hair quality goats, and group B, comprising the samples from the three non high hair quality goats.

### Establishment and verification of the cDNA Library

First, the total RNA was extracted from the skin samples and tested for integrity. Then, a cDNA library was established and verified. The Illumina HiSeq^TM^2000 system was used to sequence the transcript library. The three skin samples from the goats with the high hair quality traits were mixed for sample A, and the three skin samples from the goats with the non high hair quality traits were mixed for sample B. The samples were sequenced by computer, and the original sequencing data was stored in the FASTQ format. The sequencing work was performed by Shanghai Paisennuo Biotechnology Co. LTD. After the quality analysis of the original data, the low-quality sequences were removed, and the remaining high-quality sequences were analyzed further.

### Data processing

The filtered sequences were spliced into transcripts from scratch (*de novo* assembly) using the Trinity software (Broad Institute). Then, each transcript were compared to the sequences in NR Protein, SwissProt, Kyoto Encyclopedia of Genes and Genomes (KEGG), and eggNOG databases using BLAST and to the Gene Ontology (GO) and Unigene annotations using Blast2GO (https://www.blast2go.com/). The KEGG annotation was used to obtain the pathway annotations of Unigene. Next, the Unigenes were analyzed using eggNOG.

#### Unigene expression and analysis of differentially expressed genes

Unigene expression was calculated by the Reads Per Kilobase per Million reads (RPKM) method[17] using the following formula: RPKM = 10^9^/NL. Where RPKM is the gene expression, C is the number of reads mapped to the gene, N is the total number of mapped reads, and L is the number of bases in the gene.

#### GO functional categories enriched with differentially expressed genes

GO is the international standard for the classification of gene functions. GO annotation includes entries in three main categories: Molecular Function, Cellular Component, and Biological Process. The GO annotations were obtained for each differentially expressed gene. The annotations were then analyzed using the WEGO software[18] to get a p-value for each GO entry. If the p-value was less than 0.5, then the GO entry was considered to be significantly enriched with differentially expressed genes.

#### Metabolic pathways enriched with differentially expressed genes

The KEGG database provides data and comments about metabolic pathways, allowing a visual representation of a gene’s location in a metabolic pathway. The differentially expressed genes were compared to the KEGG database. According to the software calculation, get the p-value. If the p-value for a given metabolic pathway was less than 0.5, then the pathway was considered to be significantly enriched with differentially expressed genes.

### Validation of the transcriptome database

After the total RNA was extracted from the skin tissue, the cDNA first chain was synthesized by reverse transcription using the FastQuant RT Kit (First-strand of cDNA synthesis kit, TianGen Biotech(Beijing)CO.,LTD). The primer specificity was then detected.

#### Primer design and synthesis

Using the primer design program Primer Premier 5 and the sequence information in GenBank for *AOC3*, *DUSP1*, *VCL*, *WNK1*, *IGFBP*, and *S100A7*, fluorescence quantitative primers were obtained. The specificity of the primers was tested by Primer-BLAST. Primers for the internal gene sequences of goat *GAPDH* were obtained from a previous study by Chen Li[19]. All the primers were synthesized by Shanghai Sangon Biological Co., LTD. Table 1 shows the information for the primer pairs.

**Table 1:** The primer pairs.

Gene	Accession number	Primer sequence (5′-3′)	Product size	Annealing temperature (°C)
*AOC3*	XM_005693871.1	F:ATCCAGACGCTGGCTGTGAC	196	60
		R:TGTTGGGAATGTCCTCTGCAT		
*DUSP1*	XM_005694581.1	F:TTTTCTGCTTCCTACCCGGAG	140	60
		R:GGGCCACCCTGATCGTAGAG		
*VCL*	XM_005699224.1	F:ATTCCCTGAGCAGAAAGCCG	108	59
		R:ATGATGTCATTGCCCTTGCTG		
*WNK1*	XM_005681041.1	F:AGAGATGCGTTTGTGGAGCA	153	61
		R:TTCCAATTTTTGGGCGCCTG		
*IGFBP1*	NM_001285763.1	F:GCGATGAGGCCACAGATACA	121	60
		R:TCCTCACTGGACTCGGTCAT		
*S100A7*	XM_005677509.1	F:CTAAGCTGGAGCAGGCCATT	142	60
		R:CCCCTTTTCTCACAGGCACT		
*GAPDH*	XM_005680968. 1	F:GCAAGTTCCACGGCACAG	249	59
		R:GGTTCACGCCCATCACAA		

#### PCR amplification products

Template cDNA and 2×Taq PCR MasterMix were used to synthesize seven genes up stream and downstream of the primers. The amplification system consisted of: 1 μL cDNA template, 1 μL forward primer (10 μM), 1 μL reverse primer (10 μM), 10 μL 2×Taq PCR MasterMix, and RNase-Free ddH2O added to make a total volume of 20 μL.

The response procedures were: 3 min at 94°C; 30 cycles of 30 s at 94°C, 30 s at 55°C, and 1 min at 72°C; and 5 min extension at 72°C. The reaction product was stored at -20°C.

Then, agarose gel electrophoresis and fluorescence quantitative PCR were performed. The 7500 Software v2.0.6 of the ABI7500 fluorescence quantitative PCR Software was used for data analysis. The 2^-ΔΔC^ method was used to calculate the relative expression of gene in triplicate.We used SPSS version 15.0 (SPSS, Inc., Chicago, IL, USA) for identify significant differences.

#### Screening of genes important for high-quality brush hair

The differentially expressed genes in the skin samples were determined from the high-quality hair and non high quality hair goats based on the standard of two folds change, and screened by the significant GO functional enrichment and metabolic pathway enrichment that might be important for traits related to high-quality brush hair.

## Results

### RNA concentration and integrity testing

**Fig 1:**
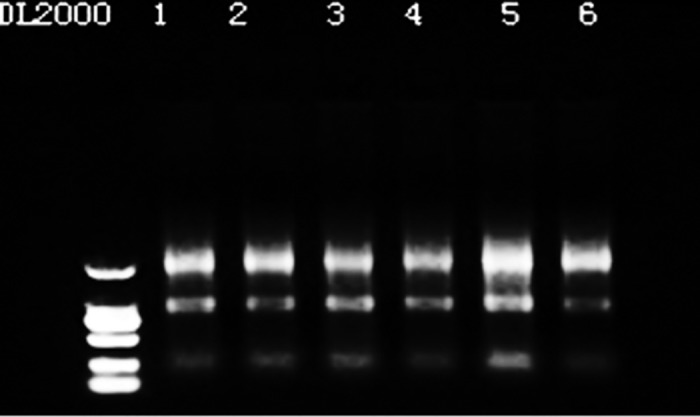
RNA sample integrity. Note: Lanes 1–6 are samples A1, A2, A3, B1, B2, and B3, respectively.

The result is shown in Fig 1.

The RNA sample concentrations’ result show as Table 2.

**Table 2:** RNA sample concentrations.

Sample Number	Sample Name	Concentration(ng/μL)	260/280	Volume (μL)	Total (μg)
1	A1	804.2	1.69	35	28.1
2	A2	673.6	1.76	35	23.6
3	A3	1,151.0	1.72	35	40.3
4	B1	1,434.2	1.72	35	50.2
5	B2	1,742.2	1.77	35	61.0
6	B3	1827.0	1.75	35	63.9

Follow-up experiments were performed after the RNA quality was tested to meet the requirements for subsequent sequencing-library construction.

### Transcriptome sequence assembly and gene functional annotation

#### Results of *de novo* assembly

The results of *de novo* assembly show as Table 3.

**Table 3:** Results of *de novo* assembly.

Assembly	Quantity	Total length (bp)	Average length (bp)	N50
Contig	485,494	110,239,434	227	236
Transcript	145,400	78,344,273	539	729
Unigene	32,812	28,840,794	879	1,387

N50:Sort the Contigs, Transcripts, and Unigenes from long to short, starting from the first series added today. The length of the Contig, Transcript, or Unigene is when the total base number reaches 50% of the total bases.

#### Gene functional annotation

The comparison to the NR, GO, KEGG, SwissProt, and eggNOG databases resulted in hits to 32,812 genes in the NR database, 23,928 genes in the GO database, 12,703 genes in the KEGG database, 28,768 genes in the SwissProt database, and 29,337 genes in the eggNOG database.

#### Unigene eggNOG analysis

**Fig 2:**
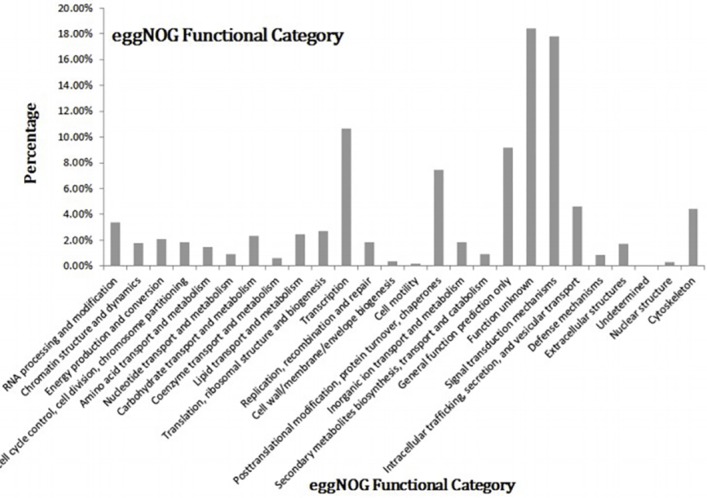
eggNOG functional categories.

The eggNOG database had statistics for 29,337 of the genes in the skin samples divided into 25 functional categories. The most highly enriched category was Signal Transduction Mechanisms, followed by the Transcription and General Function categories (Fig 2).

### Differential gene expression

Two hundred ninety-five genes were differentially expressed between the high hair quality and low hair quality goats. In the high hair quality goats, 132 genes were up-regulated, 62 genes were down-regulated, 101 genes were expressed specifically. Table 4 shows the top 20 up-regulated genes in the high hair quality goats.

**Table 4:** Top 20 up-regulated genes in the goats with type III brush hair traits.

Gene name	Gene ID	Chromosome ID	Length	High-Quality RPKM	Non High-Quality RPKM	log2Fold Change
LECT2	102180390	chr 7	668	42.0333	0.0746	8.64
apolipoprotein A-I	102179867	chr 15	1,323	188.0913	0.4143	8.32
FGG	102184178	chr 17	1,522	22.4539	0.0654	7.92
HBS1L	102178359	chr 9	816	18.4728	0.0610	7.74
VCL	102188740	chr 28	1,588	4.6669	0.0157	7.71
MAP3K1	102187530	chr 20	1,444	8.7731	0.0345	7.49
FGB	102184929	chr 17	1,989	38.5539	0.1753	7.28
DUSP6	102172327	chr 5	302	29.8185	0.1650	6.99
PAN3	102188509	chr 12	670	12.5222	0.0743	6.89
Warm-temperature-acclimation-related 65-kDa protein	100049497	chr 2	1,392	5.9871	0.0358	6.89
F2	102185018	chr 15	1,944	8.3007	0.0513	6.84
TNNT3	100861247	chr 29	777	10.2939	0.0641	6.83
WNK1	102172797	chr 5	796	9.9779	0.0626	6.82
PDZRN3	102169341	chr 22	1,117	9.3889	0.0669	6.63
ZNF470	102189614	chr 18	1,094	12.2704	0.0911	6.57
DUSP1	102180080	chr 20	428	31.1028	0.2329	6.56
TSNAX	102190379	chr 28	1,040	10.6487	0.0958	6.29
RHOT1	102179131	chr 19	1,178	9.2113	0.0846	6.27
PRPF38B	102187281	chr 3	395	23.8599	0.2523	6.06
40S ribosomal protein S14	102186113	chr 7	502	18.4399	0.1985	6.04

Note: In the NCBI database, the warm-temperature-acclimation-related 65-kDa protein gene is not annotated in Capra hircus (goat). Using BLAST, we found the wap65-like gene (100049497) in Scombermorus niphonius (Japanese Spanish mackerel), which is 97% identical to the warm-temperature-acclimation-related 65-kDa protein gene(S1 File). Therefore, we used the gene and chromosome IDs of wap65-like in Table 4.

### GO functional enrichment of differentially expressed genes

Table 5 shows the significant GO functional enrichment of the differentially expressed genes.

**Table 5:** GO functional enrichment with differentially expressed genes.

Main GO Entry	Second GO Entry	Number of Differentially Expressed Genes (%)	P-value
Cellular Component	Extracellular region	29 (9.83%)	0.012928
	Cytoplasm	147 (49.83%)	0.047668
Molecular Function	Structural molecule activity	48 (16.27%)	1.39E-18
	RNA binding	28 (9.49%)	0.002263
Biological Process	Translation	37 (12.54%)	5.14E-13
	Biosynthetic process	98 (33.22%)	0.013509
	Ribosome biogenesis	7 (2.37%)	0.016254
	mRNA processing	13 (4.40%)	0.02431
	Helicase activity	9 (3.05%)	0.026046
	Peptidase activity	22 (7.46%)	0.026575
	Generation of precursor metabolites and energy	9 (3.05%)	0.04366

### KEGG pathway enrichment with differentially expressed genes

**Fig 3:**
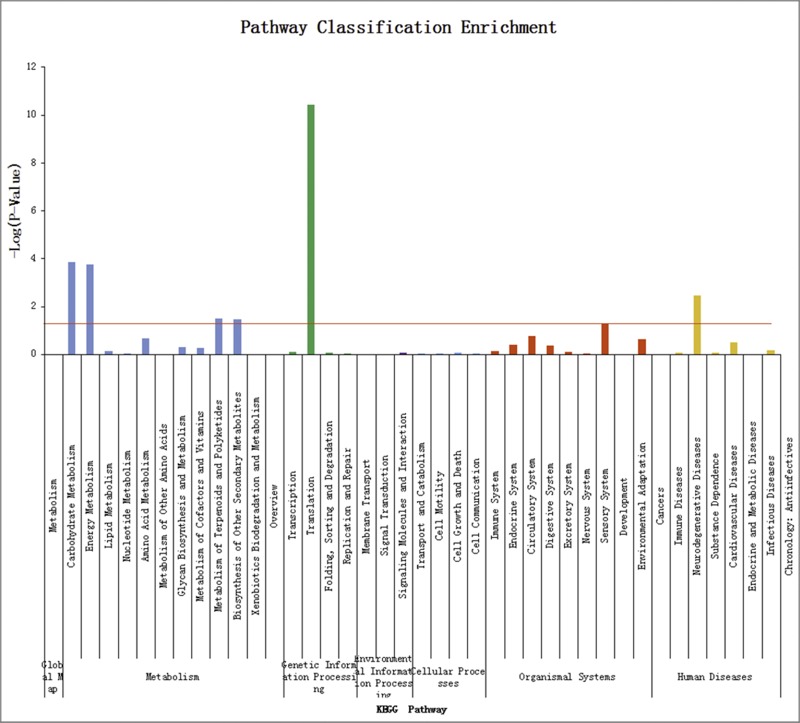
Pathway enrichment with differentially expressed genes.

KEGG pathway analysis for significant enrichment with differentially expressed genes can predict involvement in biological function. The 295 differentially expressed genes were involved in 34 signaling pathways (Fig 3). Table 6 shows the KEGG pathways that were significantly enriched with differentially expressed genes. The KEGG pathway enrichment can show how the differentially expressed genes are involved in major functional and signal transduction pathways.

**Table 6:** KEGG pathway enrichment with differentially expressed genes.

KEGG pathway	Number of differentially expressed genes (%)	p-value
Carbohydrate Metabolism	23 (7.79%)	1.41E-04
Energy Metabolism	15 (5.08%)	1.75E-04
Metabolism of Terpenoids and Polyketides	3 (1.02%)	3.17E-02
Biosynthesis of Other Secondary Metabolites	3 (1.02%)	3.49E-02
Translation	36 (12.20%)	3.85E-11
Neurodegenerative Diseases	21 (7.12%)	3.55E-03

### Important genes and pathways affecting type III hair growth

**Fig 4:**
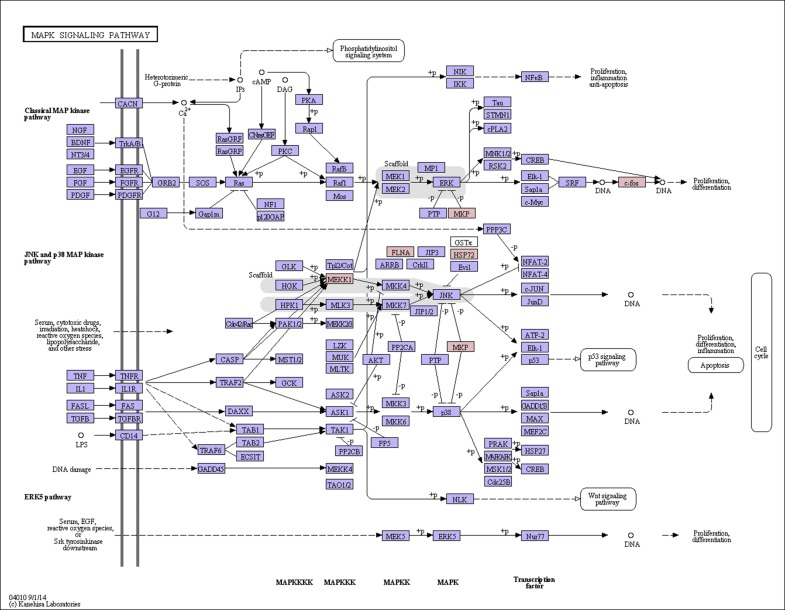
The MAPK pathway. Note: red tags showed the up-regulated genes, green tags showed the down-regulated genes.

The top 20 differentially expressed genes that were up-regulated in the high-quality hair goats were conducted for the metabolic pathway analysis, of which three differentially expressed genes (*MAP3K1*, *DUSP1* and *DUSP6*) were associated with the MAPK pathway, suggesting that the MAPK pathway plays an important role in the formation of type III hair. Furthermore, the MAPK signal pathway had totally six differentially expressed genes, with five up-regulated genes: *MAP3K1*, *DUSP1*, *DUSP6*, *FOS* and *HSPA1*, *and one* down-regulated gene: *FLNA* (Fig 4).

### Fluorescence quantitative results

#### Agarose gel electrophoresis of PCR products

**Fig 5:**
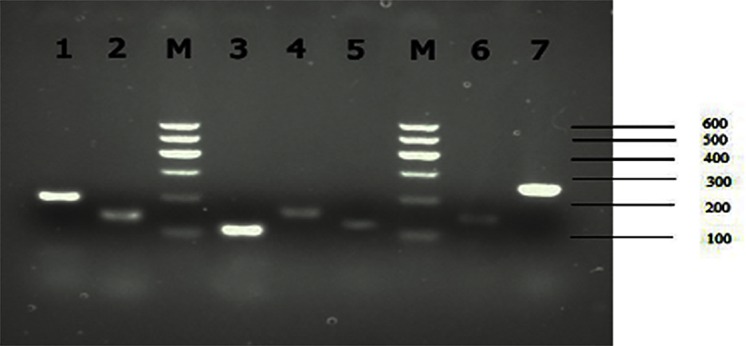
Agarose gel electrophoresis of the PCR products. Note: M: Marker I, 1: *AOC3*, 2: *DUSP1*, 3: *VCL*, 4: *WNK1*, 5: *IGFBP1*, 6: *GAPDH*, 7: *S100A7*

Agarose gel electrophoresis of the PCR products showed that all of the PCR products had a single band, indicating that the primer specificity, PCR reaction system, and PCR reaction program were satisfactory (Fig 5). Follow-up tests were therefore carried out.

#### Gene dissolution curve

**Fig 6:**
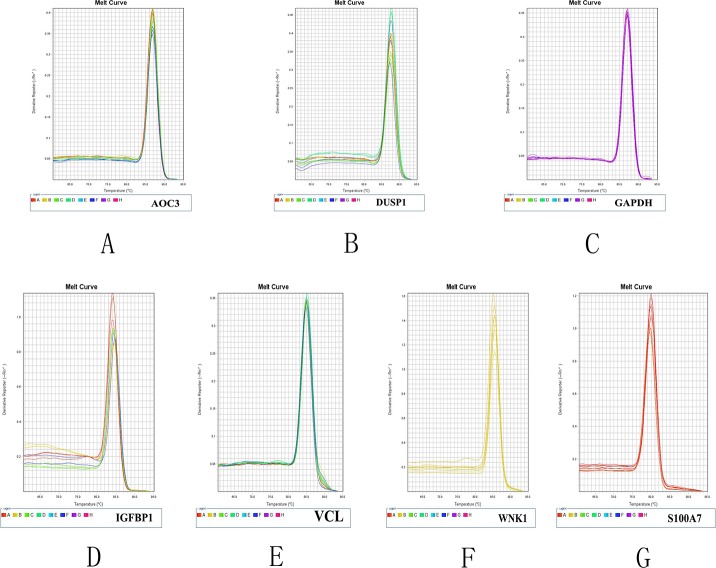
RT-qPCR dissociation curves of seven genes. Note: A is *AOC3*, B is *DUSP1*, C is *GAPDH*, D is *IGFBP*, E is *VCL*, F is *WNK1*, and G is *S100A7*.

After amplification, the dissolution curves of *AOC3*, *DUSP1*, *GAPDH*, *IGFBP*, *VCL*, *WNK1*, and *S100A7* were analyzed (Fig 6). The dissolution curves each had a single peak, indicating that the specificity of the PCR products was good and that primer-dimers and non-specific PCR amplification did not occur.

#### Relative gene expression

**Fig 7:**
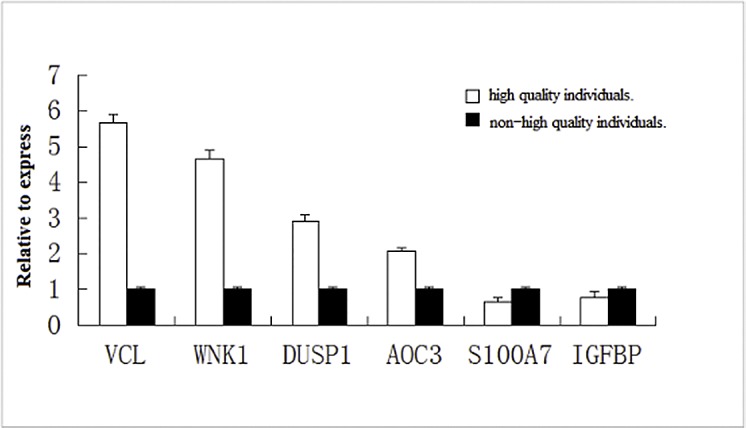
The relative expression of six genes.

Assuming that the expression levels of *VCL*, *WNK1*, *DUSP1*, *AOC3*, *IGFBP*, and *S100A7* were equal to 1 in the non high hair quality goats, the relative expression levels of those genes in the high hair quality goats were 5.67±0.25, 4.65±0.25, 2.92±0.18, 2.07±0.11, 0.78±0.16, and 0.66±0.13, respectively. The relative expression levels of *VCL*, *WNK1*, *DUSP1*, and *AOC3* were significantly higher in the high hair quality goats than in the non high hair quality goats (*P*<0.05). The relative expression levels of *IGFBP* and *S100A7* were significantly lower in the high hair quality goats than in the non high hair quality goats (*P*<0.05). The relative expression trends were consistent with the results of transcription, indicating that the results of transcription were reliable, as shown in Fig 7.

## Discussion

The RNA-Seq technique has been applied to many species and fields, such as Capra hircus[20], mice[17], and so on. It can quickly, comprehensively, and accurately detect the expression of all the genes in a sample over a certain period of time[21]. In this study, the transcriptomes of Yangtze River Delta White Goats with and without specific traits for high-quality brush hair were sequenced and analyzed. All of the Unigene annotation information for the respective samples was obtained. Analysis of the expression of all the genes in the samples identified 295 differentially expressed genes. In the goats with the high hair quality traits, 132 genes were up-regulated, 62 genes were down-regulated, and 101 genes were specifically expressed. To verify the accuracy of the transcriptome database after high-throughput sequencing, most researchers have used real-time PCR technology[22–25]. Six differentially expressed genes, including four up-regulated genes and two down-regulated genes, were randomly selected in this study. The results of those six differentially expressed genes indicated that the sequencing results were reliable.

The GO functional enrichment analysis of differentially expressed genes helps to describe the biological functions of genes of interest[26–28]. The GO analysis of the 295 differentially expressed genes resulted in 11 significantly enriched GO entries. In the Molecular Function category, the differentially expressed genes significantly enriched the Structural Molecule Activity and RNA Binding entries. In the Cell Components category, the Extracellular Region and Cytoplasm entries were significantly enriched. In the Biological Process category, the Translation, Biosynthetic Process, mRNA Processing, and Peptidase Activity entries were enriched.

The KEGG pathway analysis showed significant enrichment by the 295 differentially expressed genes in the Carbohydrate Metabolism, Energy Metabolism, and Terpenoid and Polyketone Compounds pathways, which accounted for 14% of the total differentially expressed genes. The differentially expressed genes accounted for 12% of the total differentially expressed genes. Those correlations are higher when combined with GO analysis, the traits and metabolism, and the copy and translation of the genes in the Yangtze River Delta White Goats with the high-quality hair traits. It can be further speculated that the traits could be associated with cell proliferation and differentiation.

The morphology and structure of hair follicles, the parts of the skin that produce hair, are very complex. Hair follicle development is a finely-regulated process, and this process involves a large number of genes and pathways. Studies have shown that the MAPK signaling pathway is the main participants in hair follicle growth. The MAPK pathway plays an important role in biological functions such as cell proliferation and differentiation[29–31]. The MAPK signaling pathway also play an important role in the periodic development of hair follicles, which can promote the proliferation and differentiation of hair-follicle stem cells when it activated[32–33]. MAPK is one of the important signals mediating the cellular biological response system and is common in mammals. The MAPK pathway exerts its physiological and pathological regulation via the phosphorylation of substrate. The substrates of MAPK include transcription factors, intracellular protein kinases, and skeleton-related proteins, which highlights the diversity and complexity of MAPK functions[34]. At present, there are four known MAPK signal-transduction pathways in mammalian cells: EKR (extracellular-regulated protein kinase), JNK/SAPK (c-Jun N-terminal kinase/stress-activated protein kinase), p38, and ERK5/BMK1[35].

The transcriptome sequencing results showed that three of the top 20 up-regulated differentially expressed genes, *MAP3K1*, *DUSP6* and *DUSP1*, were involved in the MAPK signaling pathway, demonstrating that the MAPK signaling pathway likely plays an important role in the formation of type III hair in the Yangtze River Delta White Goat.

*MAP3K1* was one of six differentially expressed genes in the MAPK signaling pathway. MEKK1 is the expressed protein of *MAP3K1* and is an important node in the MAPK signaling pathway. That pathway can activate c-JUN, which enhances the transcriptional activity of transcription factor AP-1, thereby regulating cell migration and the formation of actin protein fibers[36]. The current research conducted differential proteomics analysis on high quality brush hair trait in Yangtze River Delta White Goat with the results showing that β-actin interacts with keratin to participate in the intracellular transport, cell shape, movement and division of hair follicle development related cells, which might play an important role in the process for promoting hair follicle development of type III hair[37]. Actin can affect the traits of type III hair, and MAP3K1 can regulate the formation of actin protein fibers; therefore, we believe that MAP3K1 is important in determining the traits of type III hair.

The dual-specificity phosphatase (DUSP) family proteins can regulate the activity of the MAPK pathway by dephosphorylation of key pathway proteins. This family includes *DUSP1* and *DUSP6*. *DUSP1* is also called MAPK phosphatase 1. It mainly catalyzes the hydrolysis of the phosphate group in the specific sequence TxY of the activated MAPK family members (p38, JNK, ERK) in the cell, thereby inhibiting the activity of the phosphoric acid group. The activation of MAPK can induce the expression of *DUSP1*. It is an important negative feedback regulation mechanism in the MAPK signaling pathway[38–39]. *DUSP6* is also called *MKP-3* (MAP kinase phosphatases 3). Some studies have shown that *DUSP6* plays an important physiological regulatory function in the MAPK pathway[40–41]. *DUSP6* can be combined with ERK2, and it can make ERK2 inactivation by the method of negative feedback control to regulate the activity of the MAPK pathway. *DUSP1* and *DUSP6* regulate the activity of the MAPK pathway, which is closely related to the development of hair follicles. For this reason, we suspect *DUSP1* and *DUSP6* may also be key genes that affect the quality of type III hair. At present, there have been many studies of *DUSP1* and *DUSP6* in cancer, but far fewer studies on the relationship between these proteins and the development of hair follicles. We will do more in-depth research on these genes in the future, and we hope these studies will enable a clearer understanding of their function in hair follicle development.

Gene function is not independent. Upregulation or downregulation of gene expression may affect the expression of several upstream or downstream genes, thus further altering gene expression patterns and the formation of some traits. We believe that specific expression patterns of these differentially expressed genes may affect the formation of type III hair traits.

To sum up, we think that *MAP3K1*, *DUSP1*, *DUSP6* and MAPK pathway likely played an important role in the formation of the type III hair in the Yangtze River Delta White Goat.

## Supporting Information

S1 File The result of blast in the warm-temperature-acclimation-related 65-kDa protein gene. (PDF)

## References

[1] National livestock and Poultry Genetic Resources Committee, Chinese livestock and poultry genetic resources. China Agriculture Press 2011.

[2] Zhao YZ. Chinese sheep breeding. China Agriculture Press 2013.

[3] Li YJ, Huang YH. The production of wool goat and writing brush in our country. China herbivores. 2005;25(1):44–46.

[4] Pan XG, Jiang DM, Shen LS, Qiu BA, Yuan MZ, & Lu HS. Observation on the position of the goat's writing hair. Chinese Journal of Animal Science. 1985;(2):38.

[5] Huang YH, He SQ, Shan ZD, Chen YY. Study on the structure of hair fiebre of Haimen goats for writing brush. Journal of jiangsu agricultural college. 1994;16(3):51–53.

[6] Li SW, Ouyang HS, Rogers GE, & Bawden CS. Characterization of the structural and molecular defects in fibres and follicles of the merino felting lustre mutant. Experimental Dermatology. 2009;18(2),134–142. 10.1111/j.1600-0625.2008.00774.x 18637126

[7] Jia ZH, Cai QH. Research Progress on the growth mechanism and nutrition of cashmere. Acta Zoonutrimenta Sinica. 1999;(11):97–102.

[8] Gat U, Dasgupta R, Degenstein L, & Fuchs E. De novo hair follicle morphogenesis and hair tumors in mice expressing a truncated beta-catenin in skin. Cell. 1998;95(5),605–14. 9845363 10.1016/s0092-8674(00)81631-1

[9] Alexeev V, Igoucheva O, Domashenko A, Cotsarelis G, & Yoon K. Localized in vivo genotypic and phenotypic correction of the albino mutationin skin by rna-dna oligonucleotide. Nature Biotechnology. 2000;18(1),43–7. 10.1038/71901 10625389

[10] Chen W, Wang H, Dong B, Dong Z, Zhou F, & Fu Y, et al Molecular cloning and expression analysis of tyrosinase gene in the skin of jining gray goat (capra hircus). Molecular & Cellular Biochemistry. 2012;366(1–2),11–20. 22407568 10.1007/s11010-012-1275-1

[11] Li GQ, Ji YC, Li Yu. The molecular mechanisms of hair follicle morphogenesis. International Journal of Dermatology and Venereology. 2004;30(01): 38–40.

[12] Chang ZL, Liang YH, Wang RJ, Zhang WG, Chai J, & Mei BJ, et al Primary Study for Gene Regulatory Network of the Skin Follicle in Cashmere Goats. China Herbivore Science. 2006;(z1):88–92.

[13] Millar S E. Molecular mechanisms regulating hair follicle development. Journal of Investigative Dermatology. 2002;118(2),216–25. 10.1046/j.0022-202x.2001.01670.x 11841536

[14] Akilli, Öztürk, Özlem, Pakula, Hubert, & Chmielowiec, et al Gab1 and mapk signaling are essential in the hair cycle and hair follicle stem cell quiescence. Cell Reports. 2015;13(3),561–572. 10.1016/j.celrep.2015.09.015 26456821

[15] Schneider M R, Werner S, Paus R, & Wolf E. Beyond wavy hairs: the epidermal growth factor receptor and its ligands in skin biology and pathology. American Journal of Pathology. 2008;173(1),14–24. 10.2353/ajpath.2008.070942 18556782 PMC2438281

[16] Xiaoyan L I, Zhang L, Wang L, Jiao W U, Song H, & Fanjing T U, et al Selection of relative gene during secondary hair follicle growth in cashmere goat. Acta Veterinaria Et Zootechnica Sinica. 2015.

[17] Mortazavi A, Williams BA, Mccue K, Schaeffer L, & Wold B. Mapping and quantifying mammalian transcriptomes by rna-seq. Nature Methods. 2008;5(7), 621–628. 10.1038/nmeth.1226 18516045 PMC13303166

[18] Ye J, Fang L, Zheng H, Zhang Y, Chen J, & Zhang Z, et al Wego: a web tool for plotting go annotations. Nucleic Acids Research. 2006;34(Web Server issue), 293–7. 10.1093/nar/gkl031 PMC1538768 16845012

[19] Li C, Wei Z, Zhan S Y, Dan L I, Li L I, & Tao Z, et al The expression stability analysis of reference genes in the different tissues and skeletal muscle of different development periods in goat. Chinese Journal of Animal & Veterinary Sciences. 2014;45(8),1228–1236.

[20] Dong Y, Xie M, Jiang Y, Xiao N, Du X, & Zhang W, et al Sequencing and automated whole-genome optical mapping of the genome of a domestic goat (capra hircus). Nature Biotechnology. 2013;31(2), 135–41. 10.1038/nbt.2478 23263233

[21] Wang Z, Gerstein M, & Snyder A M. Rna-seq: a revolutionary tool for transcriptomics. Nature Reviews Genetics. 2009;10(1), 57–63. 10.1038/nrg2484 19015660 PMC2949280

[22] Li W, Wang P, Li Y, Zhang K, Ding F, & Nie T, et al Identification of MicroRNAs in Response to Different Day Lengths in Soybean Using High-Throughput Sequencing and qRT-PCR. Plos One, 2015;10(7):e0132621 10.1371/journal.pone.0132621 26162069 PMC4498749

[23] Geng R, Yuan C, & Chen Y. Exploring differentially expressed genes by rna-seq in cashmere goat (capra hircus) skin during hair follicle development and cycling. Plos One, 2013;8(4),e62704 10.1371/journal.pone.0062704 23638136 PMC3640091

[24] Yang H, Liu J, Huang S, Guo T, Deng L, & Hua W. Selection and evaluation of novel reference genes for quantitative reverse transcription pcr (qrt-pcr) based on genome and transcriptome data in brassica napus l. Gene. 2014;538(1),113–22. 10.1016/j.gene.2013.12.057 24406618

[25] Xu Q, Zhao W, Chen Y, Tong Y, Rong G, & Huang Z, et al Transcriptome profiling of the goose (anser cygnoides) ovaries identify laying and broodiness phenotypes. Plos One. 2013;8(2),e55496 10.1371/journal.pone.0055496 23405160 PMC3566205

[26] Wei BO, Wang LE, Chao DU, Guozhang HU, Wang L, & Ying J, et al Identification of differentially expressed genes regulated by transcription factors in glioblastomas by bioinformatics analysis. Molecular Medicine Reports. 2014;11(4),2548–2554(7). 10.3892/mmr.2014.3094 25514975 PMC4337481

[27] Mills J D, Kavanagh T, Kim W S, Chen B J, Kawahara Y, & Halliday G M, et al Unique transcriptome patterns of the white and grey matter corroborate structural and functional heterogeneity in the human frontal lobe. Plos One. 2013;8(10),e78480–e78480. 10.1371/journal.pone.0078480 24194939 PMC3808538

[28] Sheng Z, Sun Y, Zhu R, Jiao N, Tang K, & Cao Z, et al Functional Cross-Talking between Differentially Expressed and Alternatively Spliced Genes in Human Liver Cancer Cells Treated with Berberine. Plos One. 2015;10(11):e0143742 10.1371/journal.pone.0143742 26606055 PMC4659683

[29] Raman M, Chen W, & Cobb MH. Differential regulation and properties of mapks. Oncogene. 2007;26(22),3100–12. 10.1038/sj.onc.1210392 17496909

[30] Efimova T, Lacelle P, Welter J F, & Eckert R L. Regulation of human involucrin promoter activity by a protein kinase c, ras, mekk1, mek3, p38/rk, ap1 signal transduction pathway. Journal of Biological Chemistry. 1998;273(38),24387–24395. 9733728 10.1074/jbc.273.38.24387

[31] Waskiewicz A J, & Cooper J A. Mitogen and stress response pathways: map kinase cascades and phosphatase regulation in mammals and yeast. Current Opinion in Cell Biology. 1995;7(6), 798–805. 8608010 10.1016/0955-0674(95)80063-8

[32] Headon DJ, & Overbeek PA. Involvement of a novel tnf receptor homologue in hair follicle induction. Nature Genetics. 1999;22(22),370–4. 10431242 10.1038/11943

[33] Lv X, Sun W, Yin J, Ni R, Su R, & Wang Q, et al An integrated analysis of microrna and mrna expression profiles to identify rna expression signatures in lambskin hair follicles in hu sheep. Plos One. 2016;11(7):e0157463 10.1371/journal.pone.0157463 27404636 PMC4942090

[34] Cargnello M, & Roux APP. Activation and function of the mapks and their substrates, the mapk-activated protein kinases. Microbiology & Molecular Biology Reviews Mmbr. 2011;75(1),50–83. 21372320 10.1128/MMBR.00031-10 PMC3063353

[35] Davis RJ. Signal transduction by the jnk group of map kinases. Cell. 2000;103(2),239–52. 11057897 10.1016/s0092-8674(00)00116-1

[36] Wu LC, Shao YX. Map3k1 Regulation of Mouse Eyelid Closure. Chinese Journal of Zoology. 2011;46(3):144–151.

[37] Yang B, Cai MY, Li YJ, Zhang H, Cheng GH, & Zhang JH, et al Proteomic analysis identifies differentially expressed proteins participating in forming type iii brush hair in yangtze river delta white goat. Genetics & Molecular Research Gmr. 2015;14(1), 323–338. 25729965 10.4238/2015.January.23.6

[38] Li L, Chen SF, & Liu Y. Map kinase phosphatase-1, a critical negative regulator of the innate immune response. International Journal of Clinical & Experimental Medicine. 2009;2(1),48–67. 19436832 PMC2680050

[39] Liu Y, Shepherd EG, & Nelin LD. Mapk phosphatases—regulating the immune response. Nature Reviews Immunology. 2007;7(3),202–12. 10.1038/nri2035 17318231

[40] Denu JM, & Dixon JE. A catalytic mechanism for the dual-specific phosphatases. Proceedings of the National Academy of Sciences of the United States of America. 1995;92(13),5910–4. 7597052 10.1073/pnas.92.13.5910 PMC41611

[41] Zhan XL, Wishart MJ, & Guan KL. Nonreceptor tyrosine phosphatases in cellular signaling: regulation of mitogen-activated protein kinases. Chemical reviews. 2001;101(8),2477–2496. 11749384 10.1021/cr000245u

