# Supplementary material for: A Screen for Key Genes and Pathways Involved in High-Quality Brush Hair in the Yangtze River Delta White Goat
Source: PLoS One. 2017 Jan 26;12(1):e0169820. doi: 10.1371/journal.pone.0169820 (PMC5268778; doi:10.1371/journal.pone.0169820)
Supplement: S1 File — (PDF) [file pone.0169820.s001.pdf]

Gene name	Gene ID	Chromosome_ID	Length	High-Quality RPKM	Non High-Quality RPKM	log2Fold Change
warm-temperature-acclimation-related 65-kDa protein	100049497	chr 2	1,392	5.9871	0.0358	6.89

Gene sequence：

CTAATAAGCAGATTCTCCCTTCTCTGACTCCTAATCCTGACATCCCATCATTG
CTGAGGTGATGGGCTCAAACAAAGCTATGTGTCTGCCCGCAGCCAGAATCA
GCACGGTGTCATAGTGGTAAAAATGTGAACCTTTAAACACTTTAATTCCCCC
TGGACCACACAGAGCAGCATCAGGGCCGTGCAAGTATGCAGGCATAGGCA
GATCTCGGGTCACGATCCTGGGCGTGGTGGTTAGGTCGACGCCATACAGCC
TTGATCCTTTGAATATGTGAACAATGTGTTCACCCGGACAGAGAAAAGCAG
CGTCCACATGTCCTTCAAAGTTGAGCTCTTCCTTCAGGGTTTTAGGGTAGC
CTTCGATCAGCGTGTAGGGAGCAGCTGCTTTATAGATGTAAACCTGATCATC
CTTAATCAAGTAGATTTTGTCAGTGTAGGAGAAGGCAGCATCCACCCCACC
AGTCACCTCCTTCCACGCCCTCGTGATTGGGAAGGCGTGGAGGCCGTCAC
GCCGGGTGTCCAGACGCATGTACATACGGCCTGCAAATAAATAGGTTTTGC
CTACATCATCAGTGGTGATGGCATCTATTTTGAGCTCACTGCATTTAGGGAT
GCTATAATGACCTCCATGGCCAAAGTTGGGGCACGACATAAAGTAATGACG
GGCGTCCTTCGGGTAGGTCCCGTTCACCTCTCCCGATACAGGGTTGAACCT
GGTGAAGTTGTGTCCGTGGAAACAGTAGTAGTGCTCCTGCCAGCGTAGAG
CGGCAGTGCAGACAGGCAGGTGGGGCCATGTCTTGATCTTCACTGTCTTCG
TTGCAATATCATAAACATGCACATCATGTCCCTTGAAGAACAGAACCGAGT
CAGTCGTGCACTCTCCTTTGGGACACTCCACAGCAGCATCAAGGTGAGCA
GGGACTCCTGGGAAGTCTTCCTGGATTCCTTTTGGATAGCCGTCCTCTAGA
GTATGGTTATAGTAGCTGAACACCTTGTCATCCAGGAAGAAATAGATGTGAT
CGTGGTCGTCTGGGTTCTCTGCGTCGTGCATACGGAAGGCGGCGTCAACGT
GGCCTATGTTGTGGTCGTCATCCAGCTCCTTGAAGAACTCGTTGGAGAGGT
GAGCTGCACCACTGAAACCCTTCCATAGGTGGCTACCTTTGAAGAAGAAA
GTTGTTCCTTTCTCGTCAGGAGCGATTGCATCAAATTCAATCCCAGCACACC
TGTCTGGCAGAGCTCCTACTGCTGCATCTTCTGGGTGCGCCGGGGCGCCGT
TAGTGAGGCAAAAAGCCAAGCACAGAAAGAGTGATCTGGTGAGCAGCTTC
ATGACGTCCAGTCCTGATGGAGACCTTGAATGTGGCTGTAGTCTAAAAGTA
GTGTCACAAGGTAA

The results of the blast by NCBI.

Sequences producing significant alignments:

Select: All None Selected:0

Alignments Download GenBank Graphics Distance tree of results

Description	Max score	Total score	Query cover	E value	Ident	Accession
Scomberomorus niphonius warm temperature acclimation related 65kDa protein 2 (Wap65-2) mRNA, complete cds	1365	1365	97%	0.0	85%	KT356863.1
TPA: Dicentrarchus labrax warm temperature acclimation protein 65-2 mRNA, complete cds	1112	1112	93%	0.0	82%	BK006868.1
Oplegnathus fasciatus warm-temperature-acclimation-related 65-kDa protein (Wap65-2) mRNA, complete cds	1072	1072	96%	0.0	81%	JQ268276.1
Lateolabrax japonicus mRNA for warm temperature acclimation protein 65-2 (wap65-2 gene)	1024	1078	83%	0.0	83%	FR828805.1
PREDICTED: Notothenia coriiceps hemopexin (hpx), mRNA	1003	1003	81%	0.0	83%	XM_010790038.1
PREDICTED: Lates calcarifer hemopexin (hpx), transcript variant X2, mRNA	979	979	81%	0.0	82%	XM_018701742.1
PREDICTED: Lates calcarifer hemopexin (hpx), transcript variant X1, mRNA	979	979	81%	0.0	82%	XM_018701741.1
Harpagifer antarcticus mRNA for warm temperature acclimation related-like 65kDa protein (wap65-2 gene)	977	977	81%	0.0	82%	AM408054.1
Miichthys miiuy mRNA for warm temperature acclimation protein 65 KDa-2 (wap65-2 gene)	913	913	74%	0.0	83%	FN811135.1
PREDICTED: Lates calcarifer hemopexin (hpx), transcript variant X3, mRNA	905	905	75%	0.0	82%	XM_018701743.1
Larimichthys crocea Wap65-2 precursor, mRNA, complete cds	896	896	80%	0.0	81%	KJ412463.1
PREDICTED: Larimichthys crocea hemopexin-like (LOC104936899), mRNA	885	885	80%	0.0	81%	XM_010752995.1
PREDICTED: Larimichthys crocea hemopexin-like (LOC104936904), mRNA	880	880	80%	0.0	81%	XM_010753000.1
Paralichthys olivaceus Wap65-2 mRNA, complete cds	798	798	75%	0.0	81%	KC521545.1
PREDICTED: Stegastes partitus hemopexin-like (LOC103370039), mRNA	785	785	87%	0.0	78%	XM_008298941.1
PREDICTED: Haplochromis burtoni hemopexin-like (LOC102307725), mRNA	741	741	82%	0.0	79%	XM_005945146.2

激活 Windows

Scomberomorus niphonius warm temperature acclimation related 65kDa protein 2 (Wap65-2) mRNA, complete cds

GenBank: KT356863.1

FASTA Graphics

Go to:

```
LOCUS       KT356863                1725 bp    mRNA    linear   VRT 01-MAY-2016
DEFINITION  Scomberomorus niphonius warm temperature acclimation related 65kDa
            protein 2 (Wap65-2) mRNA, complete cds.
ACCESSION   KT356863
VERSION     KT356863.1
KEYWORDS    .
SOURCE      Scomberomorus niphonius (Japanese Spanish mackerel)
  ORGANISM  Scomberomorus niphonius
            Eukaryota; Metazoa; Chordata; Craniata; Vertebrata; Euteleostomi;
            Actinopterygii; Neopterygii; Teleostei; Neoteleostei;
            Acanthomorphata; Pelagiaria; Scombriformes; Scombridae;
            Scomberomorus.
REFERENCE   1  (bases 1 to 1725)
  AUTHORS   Liu,J.
  TITLE     Molecular cloning of Wap65-2 gene from Scomberomorus niphonius
  JOURNAL   Unpublished
REFERENCE   2  (bases 1 to 1725)
  AUTHORS   Liu,J.
  TITLE     Direct Submission
  JOURNAL   Submitted (30-JUL-2015) College of Life Sciences, China Jiliang
            University, Xueyuan Street 258, Hangzhou, Zhejiang 0571, P.R. China
```
